# Editorial: Recent Advances on Myocardium Physiology, Volume II

**DOI:** 10.3389/fphys.2023.1170396

**Published:** 2023-03-13

**Authors:** Norio Fukuda, Henk Granzier, Shin’ichi Ishiwata, Sachio Morimoto

**Affiliations:** ^1^ Department of Cell Physiology, The Jikei University School of Medicine, Tokyo, Japan; ^2^ Department of Cellular and Molecular Medicine, University of Arizona, Tucson, AZ, United States; ^3^ Department of Physics, Faculty of Science and Engineering, Waseda University, Tokyo, Japan; ^4^ School of Health Sciences at Fukuoka, International University of Health and Welfare, Fukuoka, Japan

**Keywords:** Ca^2+^, cardiomyopathy, heart, muscle, myosin, sarcomere, titin, troponin

Myocardium has evolved to contract in a rhythmic fashion to provide blood from the heart to the body. The mechanical activities of myocardium originate in sarcomeres, composed of three filaments [i.e., thick and thin filaments, and the giant elastic protein titin (connectin)]. Cardiac researchers have developed and applied various new technologies to elucidate the in-depth mechanisms of sarcomeric functions in the heart (Fukuda et al., 2021 and related articles therein). It is now becoming clear that sarcomeres play critical roles in the processes regulating the dynamics, growth and remodeling of the heart. These exceptional technologies have provided new prospects to facilitate the development of novel drugs for intractable heart diseases. This Research Topic of *Frontiers in Physiology* is a collection of ten original research and review papers, showing the state-of-the-art research and future directions in the physiology and pathophysiology of myocardium.

Early on, contraction of cardiac myofilaments was thought to be regulated only *via* thin filament structural changes. Namely, under the relaxing condition, the troponin (Tn) and tropomyosin (Tm) complex blocks myosin binding to actin (“*off*” state). Following an increase in the intracellular Ca^2+^ concentration ([Ca^2+^]_i_), the binding of Ca^2+^ to TnC (one of the three subunits of Tn) causes displacement of Tm on thin filaments (“*on*” state), allowing myosin to interact with actin, and as a result, active force is generated (see Kobirumaki-Shimozawa et al., 2014 and references therein). Here, it is important that strongly bound cross-bridges, such as the actomyosin-ADP complex, desuppress the inhibition of Tn-Tm, synergistically with Ca^2+^, and further activate thin filaments (Kobirumaki-Shimozawa et al., 2014 and references therein). In 2010, the group of Roger Cooke made a ground-breaking discovery showing that myosin molecues can be in a state with extremely low ATP turnover rate (Stewart et al., 2010). This novel relaxed state is widely known as the “*super-relaxed state*” (SRX) (e.g., Cooke, 2011; Irving, 2017; Craig and Padrón, 2022). SRX is in equilibrium with the “*disordered-relaxed state*” (DRX) in which myosin heads are in closer proximity to thin filaments, and can readily bind to actin (e.g., Cooke, 2011; Fusi et al., 2015). Decreasing the number of myosin molecules in SRX results in an increase in the number of the heads available to produce active force (e.g., Schmid and Toepfer, 2021). It is considered that mechanical stress on thick filaments, such as myosin binding to thin filaments (Linari et al., 2015) or titin-based passive strain (Irving et al., 2011; Hessel et al., 2022), shifts the SRX-DRX equilibrium towards DRX. SRX is a biochemical and structural state, in which myosin heads interact with, and are folded back on, the thick filament backbone, thus unavailable for interacting with thin filaments. Currently, the thick filament-based regulation is the foremost Research Topic of myofilament research.

Hypertrophic cardiomyopathy (HCM) affects more than one in 500 people worldwide with high morbidity due mostly to arrhythmia, heart failure and sudden death. Kawana et al. reviewed HCM, from mutations to mechanisms and therapies. Based on rapid advances of biochemical and biophysical techniques, they have studied the molecular effects of HCM mutations on human *β*-cardiac myosin heavy chain, combining insights from clinical genetics and structural analyses of cardiac myosin. Accordingly, they concluded that HCM-causing mutations in sarcomere proteins cause hypercontractility, and that the increase in the number of myosin molecules available for interacting with actin is the primary driver. While *β*-blockers and Ca^2+^ channel blockers have been widely used to alleviate the heart’s hyperdynamic contraction for decades, their effectiveness is often poorly tolerated in the clinical setting, especially for young patients. Recently, two small molecule cardiac myosin inhibitors that shift the SRX-DRX equilibrium to SRX have been developed. One of these, mavacamten, showed improvement in the composite endpoint of exercise capacity and symptom severity in the phase III study of HCM patients, and it has recently been approved by the US Food and Drug Administration. Mavacamten is the first compound that showed a benefit for HCM in a randomized controlled trial. The other small molecule cardiac myosin inhibitor is aficamten; it is currently under evaluation in a phase III trial, and the results are awaited.

On another front, omecamtive mecarbil (OM) was developed as a first-in-class myosin activator for the treatment of heart failure in patients with reduced ejection fraction (HFrEF). Several lines of evidence suggest that OM is effective in the treatment of HFrEF by improving cardiac function without increasing [Ca^2+^]_i_. This is a favorable property as an inotropic agent because an increase in [Ca^2+^]_i_ often causes arrhythmias due to membrane depolarization coupled with Na^+^ entry *via* the Na^+^/Ca^2+^ exchanger. Nakanishi et al. demonstrated by using skinned porcine venticular and atrial muscles that OM increases Ca^2+^ sensitivity in a concentration-dependent manner in a clinically relevant range (i.e., 0.5 and 1 μM), with the effect more pronounced in ventricular muscle. The Ca^2+^-sensitizing effect of OM became less following thin filament reconstitution with fast skeletal Tn complex, and the ensuing decrease in the number of “*recruitable*” cross-bridges. Therefore, they concluded that OM’s Ca^2+^ sensitizing effect results from the strongly bound cross-bridge-dependent allosteric activation of thin filaments. Also, as pointed out by Kampourakis et al. (2018), OM may modulate the thick filament structure and destabilize SRX, resulting in an increase in the number of myosin molecules that can readily bind to thin filaments and produce active force.

Yet further efforts are needed from various angles to fully uncover how the myosin state in SRX or DRX regulates contraction and relaxation of myocardium in living conditions, especially in the beating heart *in vivo*; myosin targeted drugs will open the door to new treatments for intractable heart diseases.

Three papers have added to the recent advances in the understanding of thin filament regulations. Accumulating evidence indicates that mutations in thin filament proteins cause genetic heart diseases such as HCM, dilated cardiomyopathy (DCM) and restictive cardiomyopathy (RCM) (e.g., Ohtsuki and Morimoto, 2008). Due to the lack of accurate information on the structural properties of proteins, our knowledge on the molecular mechanisms of thin filament regulation is still insufficient, especially in the presence of mutations. Just in the last decade, cryogenic electron microscopy (cryoEM) has emerged as the most powerful technique in biological sciences to reveal atomic structures, and this technology has been used in our research field as well. By taking advantage of computational analyses (i.e., structure prediction, protein-protein docking, molecular dynamics flexible fitting and molecular dynamics simulations), Rynkiewicz et al. analyzed the TnT domain spanning the head-to-tail overlap domain of tropomyosin, and refined the published cryoEM modeled structures. They also reinterpreted the interactions between Tm and TnI showing key features that hold Tm in its sterically blocking position at low [Ca^2+^]_i_. This refined understanding of thin filament structures will provide us with many opportunities such as designing molecular interventions for genetic heart diseases.

Chalovich et al. reviewed thin filament regulations with a focus on Tn mutants, in particular the HCM-causing mutation of Δ14 of TnT that is missing the last 14C-terminal residues of cardiac TnT. It is important that removal of the basic residues in this region eliminates the inactive Ca^2+^-free state. Indeed, cardiac muscle fibers containing Δ14-TnT shows increased Ca^2+^ sensitivity by ∼0.23 pCa units with no change in maximal Ca^2+^-activated force (Nakaura et al., 1999), a finding that demonstrates that elimination of the inactive Ca^2+^-free state results in increased basal activity. It is therefore likely that the C-terminal region of TnT limits Ca^2+^ activation in myocardium. As pointed out by Chalovich et al., systematic analyses of Tn mutants, occurring naturally or post-translationally, provide us with opportunities to fully elucidate details of actin-based regulations of myocardial contraction.

Mahmud et al. designed the novel small molecule Tn activator RPI-194 that targets Tn to increase Ca^2+^ sensitivity in myocardium. By using nuclear magnetic resonance, they determined that RPI-194 binds to cardiac Tn, and stabilizes the activated complex between TnC and the switch region of TnI. RPI-194 acts as a so-called “*Ca* ^*2+*^ *sensitizer*” in that the compound at 100 μM shifts the mid-point of the force-pCa curve by ∼0.28 and ∼0.71 pCa units, respectively, in skinned rat cardiac trabeculae and slow skeletal muscle fibers. It shows cross-reactivity with skinned rat fast skeletal muscle fibers by shifting pCa_50_ by ∼0.25 pCa units. It is to be noted that RPI-194 decreases the velocity of contraction in living unloaded cardiomyocytes from mice. RPI-194 represents a new class of non-specific Tn activators that could potentially be used either to enhance cardiac muscle contractility in the setting of systolic heart failure or to enhance skeletal muscle contraction in neuromuscular disorders.

Relaxation of myocardium is the key process for effective ventricular filling, and the duration of isovolumic relaxation depends on the rate of the transition from late-systole to early-diastole. There are two important contributors to this transition; i.e., the fall of [Ca^2+^]_i_ and the detachment of cross-bridges. The increased rate of cross-bridge detachment leads to faster ventricular relaxation. Wakefield et al. investigated the effects of the hydrolytic product inorganic phosphate (Pi) on the myocardial relaxation rate when sarcomere lengthening (1%) was applied. Accordingly, they found that Pi enhanced the stretch-dependent increase in the cross-bridge detachment rate, with the magnitude becoming greater with faster stretching. Considering that Pi exists in the mM range in cardiomyocytes under physiological conditions, their finding will promote our understanding of the molecular mechanism of sarcomeric relaxation during the transition from late-systole to early-diastole.

Sarcomeres repeatedly shorten and lengthen in response to a change in [Ca^2+^]_i_ in myocytes under physiological conditions. In 2015, Shintani and colleagues found that when warmed to 37–43°C, sarcomeres exhibit rapid spontaneous sarcomeric auto-oscillations independent of [Ca^2+^]_i_ changes in neonatal rat cardiomyocytes (Shintani et al., 2015). They termed this phenomenon “*Hyperthermal Sarcomeric Oscillations* (HSOs).” HSOs are similar in characteristics to “*Spontaneous Oscillatory Contractions* (SPOCs)” that occur at partial activation of pCa ∼6.0 in both neonatal and adult myocardium (e.g., Ishiwata et al., 2011; Shintani et al., 2014; Kagemoto et al., 2018); however, the frequency of HSOs is higher than that of SPOCs. Yet it is unknown whether or not HSOs (or SPOCs) occur under physiological conditions, Shintani provides a unique idea that sarcomeric spontaneous re-lengthening mechanisms may operate in the beating heart, allowing for rapid and effective relaxation of myocardium.

Haftbaradaran Esfahani et al. provide a novel hypothesis that dynamic changes in the geometry of cardiomyocytes play a role in their plasticity and signaling, with the magnitude of the effects being greater with increased beating frequency. This hypothesis was tested experimentally by fluorescence resonance energy transfer-based imaging of the activity of Src kinase and mathematically by assuming the constant volume behavior of cardiomyocyte contraction, i.e., the length shortening is compensated by Z-disk myofilament lattice expansion and dynamic deformation of the membrane between two adjacent Z-disks. Their experiments demonstrated higher concentrations of phospho-Src at higher beat frequencies. Yet future studies need to be systematically performed; their concept of locality of the surface-to-volume ratio may advance our understanding of the membrane-mediated signaling and plasticity of myocardium in response to biomechanical stress in the heart.

Kötter and Krüger review the roles of the giant elastic protein titin, with an emphasis on the relationship between the molecule and protein quality control. Titin, the largest molecule in biology, is well known for its multiple functions such as serving as a molecular spring in the sarcomere and a molecular ruler during embryonic development (e.g., Granzier and Labeit, 2004; Fukuda et al., 2008). In myocardium, titin is involved in length-dependent activation by decreasing the distance between the thick and thin filaments *via* its passive force, thereby forming the basis of the Frank-Starling law of the heart (e.g., Granzier and Labeit, 2004; Fukuda et al., 2008). Aside from these traditional roles, recent evidence indicates that titin plays a critical role in cellular signaling (e.g., Krüger and Linke, 2011; Linke and Hamdani, 2014; van der Pijl et al., 2018). In this review article, they discuss how proteasome, autophagy, heat shock proteins and proteases are involved in the protection and degradation of titin in myocardium, as well as in skeletal muscle. Although future studies are needed to fully uncover the fine-tuned sarcomere turnover processes, the comprehensive work by Kötter and Krüger will be useful for researchers in the field to understand the physiological roles of titin, and various muscular disease states due to the imbalance of the quality control of protein.

One paper introduces a technological advancement of the non-invasive analysis of *in vivo* cardiac function. In some areas of cardiac research, clinical approaches have surpassed research methods, allowing for in-depth analysis of the various tiers of heart function. Sturgill et al. reviewed the clinical usefulness of speckle tracking echocardiography (STE) for the quantification of cardiac function. STE uses B-mode imaging which records a video clip of the entire heart wall. Therefore, as an average measurement, STE provides data points along the entire heart wall rather than merely two from M-mode imaging. STE allows us to obtain displacement, velocity, strain and strain rate in radial, longitudinal and circumferential axes of the heart. STE is useful in basic research; in particular, further improvement of spatial and temporal resolutions will allow for more accurate analyses of the *in vivo* assessment of all tiers of cardiovascular performance in small rodents like mice, including left ventricular systolic and diastolic function and contractility.

In summary, this Research Topic highlights the considerable advancements cardiac researchers have made since the turn of the 21st century. We must move forward to further elucidate the physiology and pathophysiology of myocardium not only by taking advantage of the current technologies but also by developing newer advanced technologies. In doing so, all the contributions made by the authors in the Research Topic will be integrated for the prevention, diagnosis and treatment of disorders of the heart.

## Abbreviations

[Ca^2+^]_i_, intracellular Ca^2+^ concentration; cryoEM, cryogenic electron microscopy; DCM, dilated cardiomyopathy; DRX, disordered-relaxed state; HCM, hypertrophied cardiomyopathy; HFrEF, heart failure with reduced ejection fraction; OM, omecamtive mecarbil; Pi, inorganic phosphate; SRX, super-relaxed state; STE, speckle tracking echocardiography; Tm, tropomyosin; Tn, troponin.
